# Dysregulation of SELENOI Is Associated with TDP-43 Neuropathology in Amyotrophic Lateral Sclerosis

**DOI:** 10.3390/cells14181457

**Published:** 2025-09-17

**Authors:** Finula I. Isik, Jasmin Galper, Russell Pickford, Nicolas Dzamko, YuHong Fu, Woojin Scott Kim

**Affiliations:** 1 Brain and Mind Centre, The University of Sydney, Sydney, NSW 2006, Australia; 2 School of Medical Sciences, The University of Sydney, Sydney, NSW 2006, Australia; 3 Bioanalytical Mass Spectrometry Facility, University of New South Wales, Sydney, NSW 2052, Australia; 4 School of Biomedical Sciences, University of New South Wales, Sydney, NSW 2052, Australia

**Keywords:** amyotrophic lateral sclerosis, motor neuron disease, phosphatidylethanolmine, SELENOI, TDP-43, motor cortex

## Abstract

Amyotrophic lateral sclerosis (ALS), also known as motor neuron disease, is characterized by progressive degeneration of motor neurons and accumulation of TAR DNA-binding protein 43 (TDP-43) in the brain. Increasing evidence indicates that aberration in lipid synthesis or regulation underlies neuronal dysfunction and degeneration. Phosphatidylethanolmine (PE) is an abundant phospholipid in the brain and is synthesized by the SELENOI gene. SELENOI is important in motor neuron development and function, as demonstrated in hereditary spastic paraplegia, a neurological disorder in which SELENOI is mutated. Despite this, virtually nothing was known about SELENOI in the context of ALS neuropathology. We therefore undertook a comprehensive assessment of PE in ALS brain tissues, using sophisticated liquid chromatography-mass spectrometry, and investigated how SELENOI regulates TDP-43 expression. PE levels were significantly decreased in the disease-affected motor cortex of ALS compared to controls and were inversely associated with disease duration. In contrast, PE levels were unaltered in the disease-unaffected cerebellum. Consistent with this, SELENOI expression was dysregulated only in the motor cortex of ALS. The correlation between SELENOI and TDP-43 was also lost in the motor cortex of ALS. A knockdown of SELENOI expression in neuronal cells caused an upregulation of TDP-43 expression. When put together, these results suggest that SELENOI dysregulation may contribute to TDP-43 pathology in ALS brain. Our study has provided new insights into an unrecognized pathway in ALS brain and revealed new targets for controlling TDP-43 pathology in ALS brain.

## 1. Introduction

Amyotrophic lateral sclerosis (ALS) is a rapidly-progressing neurodegenerative disease affecting motor neurons in the brain and spinal cord that results in the loss of muscle control, spasticity and weakness in the limbs. ALS is characterized by motor and sensory nerve and muscle pathology, resulting in inability to move, speak, swallow and breathe properly. The primary region of the brain affected in ALS is the motor cortex. The majority

(~90%) of ALS cases are sporadic, with the remaining ~10% associated with variations in >40 genes [1]. ALS is a heterogeneous group of disorders that is neuropathologically characterized by the accumulation of TAR DNA-binding protein 43 (TDP-43) in many cases, which is encoded by the TARDBP gene [2,3]. TDP-43 is a transcriptional factor that binds to both DNA and RNA to regulate transcriptional and translational processes [4].

An area of ALS research that is emerging is the understanding of the contribution of lipid dysregulation in ALS pathology, and in particular, TDP-43 neuropathology. Lipid dysregulation in multiple pathways is prevalent in ALS blood, with evidence that phospholipid regulates TARDBP expression [5]. Similar to other pathogenic proteins, growing evidence indicates that lipids play a role in the aggregation process of TDP-43 [6]. Lipids are a chemically diverse group of organic compounds that include fatty acids, cholesterol, triglycerides and phospholipids among many others. Phosphatidylethanolamine (PE) is a phospholipid that is highly enriched in the brain. PE is synthesized by selenoprotein I (SELENOI; also known as EPT1), a phosphotransferase enzyme in the Kennedy synthesis pathway. PE and its derivative, plasmenyl-PE (also known as plasmalogen), are important for the development and function of motor neurons [7]. They play roles in protecting neurons from the damaging effects of neuroinflammation and reactive oxygen species and facilitating cell recovery [8,9]. Evidence that demonstrates the possible importance of PE in ALS neuropathology comes from a study of a *Drosophila melanogaster* model of ALS. In these flies, PE levels were significantly decreased in the brain with concomitant neuronal deterioration [10], suggestive of a common pathology in broad ALS models.

SELENOI is also highly expressed in the brain [11] and indispensable for neurodevelopment and neuroprotection [12], as demonstrated in humans with loss-of-function mutations in the SELENOI gene. These rare mutations cause hereditary spastic paraplegia (HSP), a heterogeneous developmental and neurological disorder. HSP is typically characterized by progressive degeneration of motor neurons, resulting in spasticity and contraction in the lower limbs that affect muscular movement and walking [13]. In mouse studies, global knockout of the SELENOI gene is embryonic lethal [14]. However, conditional knockout of SELENOI in the brain alters brain development, architecture and function, resulting in a significant impairment to motor coordination and weakness in the hindlimbs [15].

Despite the importance of PE and SELENOI in motor neuron function, very little is known about PE, and virtually nothing was known about SELENOI in the context of ALS neuropathology. In this study, we undertook a comprehensive assessment of PE and SELENOI in different regions of ALS brain and investigated how changes in SELENOI affect TDP-43. We reveal a new lipid dysregulation pathway in ALS brain.

## 2. Materials and Methods

### 2.1. Human Brain Tissues

Frozen postmortem brain tissue samples were obtained from NSW Brain Tissue Resource Centre and Sydney Brain Bank (Sydney, Australia). Ethical approval was from University of New South Wales Human Research Ethics Advisory. Frozen samples from the motor cortex and the cerebellum from 12 sporadic ALS with TDP-43 pathology, as determined by the current consensus diagnostic criteria and the presence of TDP-43 deposits in brain tissues as measured by immunohistochemistry [16], and 10 controls without neurological, psychiatric or neuropathological diagnoses, and no evidence of TDP-43 deposits in brain tissues, were used in this study. The demographic information on the human tissues is provided in Table 1.

### 2.2. Chemicals and Materials

Lipids were extracted using methyl-t-butyl ether, methanol (Sigma Aldrich, St. Louis, MO, USA) and ultrapure water (Millipore). All solvents used were HPLC grade or higher. Glasswares (Sigma and VWR) were used wherever possible to minimize plastic contamination of samples. Lipid internal standards (Avanti Polar Lipids Inc., Alabaster, AL, USA) include phosphatidylethanolamine (17:0), phosphatidylcholine (19:0), phosphatidylserine (17:0), phosphatidylglycerol (17:0), phosphatidic acid (17:0), phosphatidylinositol (17:0 14:1), sphingomyelin (12:0), ceramide (d18:1, 12:0), monoglyceride (17:0), diglyceride (1,3 18:0 d5), triglyceride mix d5 (cat. no. LM-6000), diglyceride mix d5 (cat. no. LM-6001), cholesteryl ester (19:0), C12 GluCer, C17 ceramide, C17 S1P, D3 C20 fatty acid, C12 C1P, C12 sulfatide, C12 LacCer, and C17 sphingosine. Lipid internal standards were prepared as a mixture at typically 10 pmol/µL in methyl-tert butyl ether and methanol (MTBE:methanol, 1:1 *v*/*v*).

### 2.3. Immunohistochemistry

Firstly, formalin-fixed, paraffin-embedded sections (10 µm) were deparaffinized in xylene and rehydrated through graded ethanol, followed by antigen retrieval with citric buffer (pH 6.0) using a pressure cooker (Aptum Bio Retriever 2100, Aptum Biologics Ltd., UK) at a peak temperature of 121°C and gradually cooled to RT. Blocking of endogenous peroxidase was achieved with 1% hydrogen peroxide in 50% ethanol. Sections were blocked with 5% normal horse serum, then incubated with TDP-43 monoclonal antibody (Cosmo Bio Ltd., cat. no. CAC-TIP-PTD-M01A, 1:2,000) at 4 °C for two nights, followed by the secondary antibody (ImmPRESS^®^-AP Horse Anti-Mouse IgG Polymer Detection Kit, Alkaline Phosphatase, cat. no. MP-5402) as per the manufacturer’s instructions. Finally, sections were then counterstained with hematoxylin and cover-slipped. Images were obtained at x20 magnification using an Olympus slide scanner (VS-200).

### 2.4. Lipid Extraction

Lipid extraction of brain tissues was done based on the Matyash method [17]. A total of 10 µL of the internal standards (10 pmol/µL) were added to 10 mg of fresh-frozen brain tissues and homogenized in methanol containing 0.01% BHT (300 µL) using a Qiagen TissueLyser (3 × 30 sec, 30 Hz cycles). The homogenates were transferred to glass tubes, as well as the methanol (430 µL) wash of the beads. To the mixture, MTBE (2.42 mL) was added, vortexed and incubated for 30 min at RT. To the mixture, 600 µL of water was added, vortexed and centrifuged at 1000× *g* for 10 min. Using a glass Pasteur pipette, the upper phase was transferred to a new glass tube. The lower phase was re-extracted using MTBE/MeOH/water (10:3:2.5). The combined extracts were dried under nitrogen gas. Dried lipid samples were reconstituted in methanol:chloroform (1:1, 100 µL) and stored at −80 °C in glass liquid chromatography-mass spectrometry (LC-MS) vials.

### 2.5. Liquid Chromatography—Mass Spectrometry

10 μL of lipid extracts were analyzed using a Q-Exactive HF Mass Spectrometer coupled to a U3000 UPLC system (ThermoFisher Scientific). Chromatography was carried out on a Waters CSH C18 UHPLC column 2.1 × 100 mm, 1.8 μM with VanGuard guard column at 60 °C. Solvent A was 4:6 water:acetonitrile and Solvent B was 9:1 isopropanol:acetonitrile, both with 10 mM ammonium formate and 0.1% formic acid. The method of Castro-Perez et al. [18] was used for the lipid chromatography. Briefly, a 30 min gradient running from 30 to 100% of solvent B was performed, eluting lipids in order of hydrophobicity. Column eluate was directed into the electrospray ionization source of the mass spectrometer where a HESI probe was employed. Source parameters were broadly optimized on a range of lipid standards prior to the analysis. The mass spectrometer was run in data-dependent acquisition mode. A survey scan over the mass range 200–1200 at resolution 70 K was followed by 20 data-dependent MS/MS scans on the most intense ions in the survey at 15 K resolution. Dynamic exclusion was used to improve the number of ions targeted. Cycle time was approximately 1 sec. Samples were run in both negative and positive polarities. LipidSearch software 4.2.29 was used for the data analysis. Data were searched against the standard Lipidsearch database with all common mammalian lipid classes included. Abundance of lipids was obtained from peak areas for each lipid species. They were normalized between samples to correct for batch effects from the sample preparation and the LC-MS analysis, using the internal standards of the same lipid category. They were then normalized to the weight of the brain tissues used.

### 2.6. Protein Extraction and Western Blotting

Protein was extracted from fresh-frozen brain tissues (100 mg) as previously published [19]. The bicinchoninic acid assay (Pierce BCA Protein Assay Kit) was used to determine protein concentrations. Western blotting was carried out as previously described [20] using TDP-43 (Proteintech, cat. no.10782-2-AP, 1:5000), NfL antibody (Cell Signaling, cat. no. 2835S, 1:2000) and SELENOI (Sigma-Aldrich, cat. no. HPA064125, 1:1000) antibodies. Signals were detected using enhanced chemiluminescence and Gel Doc System (Bio-Rad). The blots were stripped and probed for housekeeper protein β-actin. The signal intensity was quantified using Image Lab (Bio-Rad).

### 2.7. Cell Studies

SH-SY5Y neuronal cells (ATCC, Manassas, VA) were cultured in DMEM containing 10% fetal calf serum, 1% Glutamax, 0.5% glucose, 100 IU/mL penicillin and 100 μg/mL streptomycin at 37°C in humidified air containing 5% CO_2_. In the SELENOI knockdown study, cells were cultured in 12-well plates and transfected with SELENOI siRNA (Qiagen GeneGlobe ID: SI03131345, cat. no.: 1027417) or scramble siRNA (control). The cells were harvested after 48 h and total RNA prepared for gene expression studies.

### 2.8. RNA Extraction and Quantitative PCR

TRIzol reagent (Invitrogen) was used to extract total RNA as previously described [21], which were carried out using RNase-free reagents. RNA (1 μg) was reverse transcribed into cDNA using M-MLV reverse transcriptase and random primers. qPCR assays were carried out using a Mastercycler ep realplex S and the fluorescent dye SYBR Green. Each 20 μL reaction contained 1x mastermix, 5 pmoles of primers and 1 μL of cDNA template. Amplification was carried out with 40 cycles of 94 °C for 15 s and 60 °C for 1 min. Gene expression was normalized to the geometric mean of three housekeeper genes, GAPDH (AATGAAGGGGTCATTGATGG, AAGGTGAAGGTCGGAGTCAA), β-actin (GAATTCTGGCCACGGCTGCTTCCAGCT, AAGCTTTTTCGTGGATGCCACAGGACT) and PPIA (AGGGTTCCTGCTTTCACAGA, GTCTTGGCAGTGCAGATGAA). A no-template control was included for each PCR amplification assay. The level of expression for each gene was calculated using the comparative threshold cycle (Ct) value method using the formula 2^−ΔΔCt^ (where ΔΔCt = ΔCt sample − ΔCt reference).

### 2.9. Statistical Analysis

Statistical analyses were carried out as per a previous publication [21] using SPSS Statistics software version 26 (IBM, Chicago, Illinois). To determine differences in lipid levels in ALS and control groups, multivariate analyses (general linear model) were used, with post hoc statistical significance set at *p* < 0.05. All statistical analyses were covaried for age, sex and postmortem delay to normalize for any difference between ALS and control groups. GraphPad Prism 10.4.1 was used to generate the graphs. Pearson’s correlations were used to determine if changes in measurements were associated with each other with statistical significance set at *p* < 0.05.

## 3. Results

### 3.1. Phosphatidylethanolamine Is Decreased in ALS Motor Cortex

**Figure 1:**
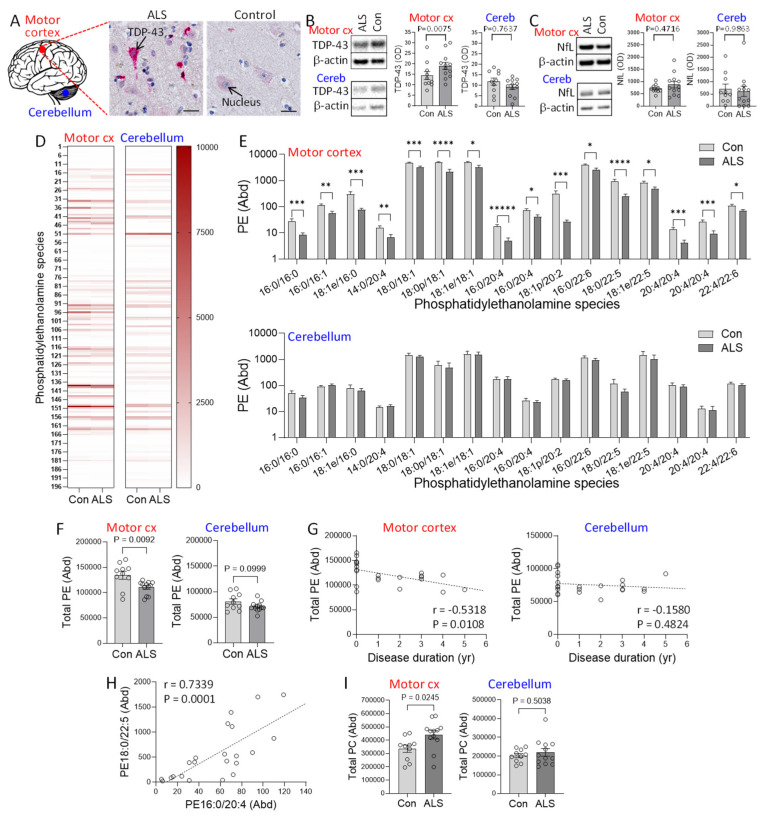
Assessment of phosphatidylethanolamine in amyotrophic lateral sclerosis (ALS) brain. (**A**) TDP-43 deposits (red staining) localized in the neurons of ALS motor cortex, counterstained with hematoxylin to show the cell nucleus blue. Bar = 20 µm. (**B**) TDP-43 expression in the disease-affected motor cortex and disease-unaffected cerebellum of ALS (N = 12) and controls (N = 10) as measured by western blotting and normalized with β-actin. (**C**) NfL in the motor cortex and cerebellum as measured by western blotting and normalized with β-actin. (**D**) Heat map of 196 phosphatidylethanolmine (PE) species in the two brain regions. (**E**) PE species (Abundance) that are altered in ALS compared to controls. The abundance was obtained from LC-MS peak areas relative to internal standards and normalized to the weight of the brain tissues used. *p* < 0.05 *, 0.01 **, 0.005 ***, 0.001 ****, 0.0005 *****. (**F**) Total PE (Abundance) in ALS compared to controls in motor cortex and cerebellum. (**G**) Correlation between total PE and disease duration in the two brain regions. (**H**) An example of the association among the PE species that are decreased in the motor cortex. (**I**) Total phosphatidylcholine (PC) (Abundance) in ALS compared to controls. Data represent mean and S.E.M. as error bars.

Despite the importance of phosphatidylethanolamine (PE) in neuronal development and function, very little is known about PE in the context of ALS brain. We therefore undertook a comprehensive analysis of PE in sporadic ALS (N = 12) and control (N = 10) (Table 1) using highly-sensitive liquid chromatography-mass spectrometry (LC-MS). The motor cortex, the primary region of the brain affected by ALS and with significant presence of TDP-43 deposits, and the cerebellum, a region of the brain with rare presence of TDP-43 deposits, were analyzed. All ALS cases were confirmed to have TDP-43 deposits as measured by immunohistochemistry, whereas all controls were free of TDP-43 deposits (Figure 1A). Firstly, we verified the presence of TDP-43 deposits localized in the neurons of ALS motor cortex using immunohistochemistry (Figure 1A). The level of TDP-43 was also measured by western blotting and covaried with age and sex. It was elevated in the motor cortex of ALS compared controls but unaltered in the cerebellum (Figure 1B). We also assessed neurofilament light protein (NfL) in the same tissues and found that it was not significantly altered in the motor cortex nor the cerebellum (Figure 1C), indicating neuronal integrity within these tissue structures in these samples.

196 PE species (Figure 1D) were detected by LC-MS, and the abundance of each of the species and the combined total of all PE species (total PE) were analyzed using LipidSearch software and multivariate tests covarying for age and sex. Neither age nor sex had a significant effect on PE (P = 0.2196 and 0.9755, respectively). In the motor cortex, 16 PE species were significantly decreased in ALS compared to controls, with most others decreasing non-significantly (Figure 1E). The total PE was also significantly decreased in ALS compared to controls (Figure 1F). In contrast, none of the PE species (Figure 1E) or the total PE (Figure 1F) were altered in the cerebellum. Furthermore, the total PE was inversely associated with disease duration only in the motor cortex (Figure 1G). We also observed a strong association among the PE species (Figure 1H), suggesting that the PE species were under the same regulatory control. We also assessed the phospholipid phosphatidylcholine (PC) because it was shown to be increased in the brain of animal models of HSP and ALS as a compensation for the PE decrease [10,15]. Consistent with this, we found that PC levels were increased in the motor cortex of ALS compared to controls, whereas they were unaltered in the cerebellum (Figure 1I).

### 3.2. SELENOI Expression Is Dysregulated in ALS Motor Cortex

**Figure 2:**
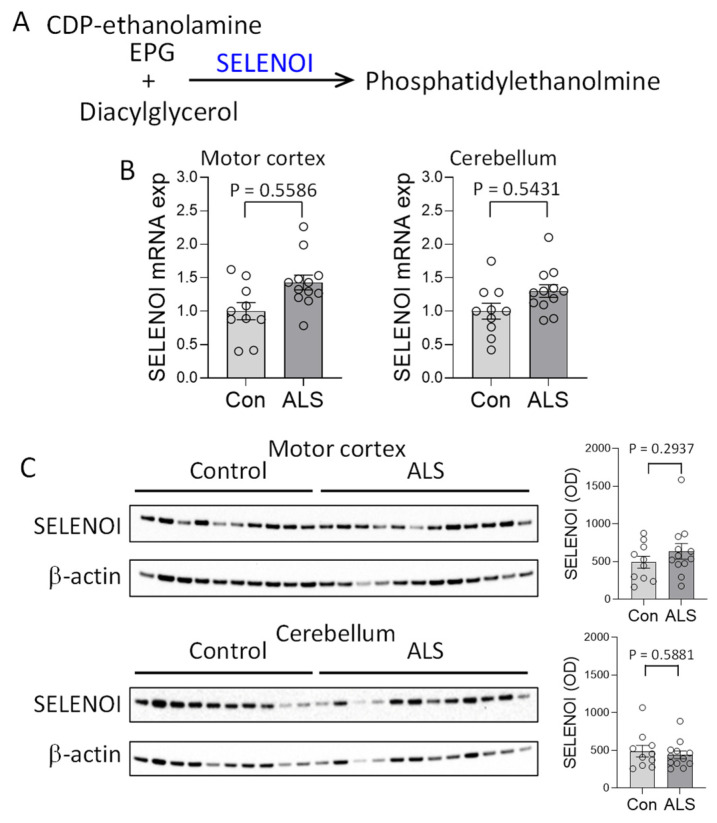
Assessment of SELENOI expression in amyotrophic lateral sclerosis (ALS) brain. (**A**) SELENOI catalyzes the transfer of the ethanolamine phosphate group (EPG) from cytidine diphosphate (CDP)-ethanolamine to diacylglycerol to form phosphatidylethanolmine (PE). (**B**) SELENOI mRNA expression in the disease-affected motor cortex and disease-unaffected cerebellum of ALS (N = 12) and controls (N = 10) as measured by qPCR, covaring for age and sex, and normalized with the geometric mean of three housekeeper genes. (**C**) SELENOI protein expression in the motor cortex and cerebellum of ALS (N = 12) and controls (N = 10) as measured by western blotting, covaring for age and sex, and normalized with β-actin. Data represent mean and S.E.M. as error bars.

To understand the possible cause of the PE decrease in ALS motor cortex, we assessed the expression of SELENOI, the gene responsible for PE synthesis (Figure 2A). Using the same tissue samples as the lipid analysis, SELENOI expression was analyzed, covaring for age and sex. SELENOI mRNA expression was unaltered in the motor cortex of ALS compared to controls (Figure 2B). SELENOI protein expression was also unaltered (Figure 2C). These results were surprising because a decrease in PE would have caused an upregulation of SELENOI expression as a feedback response [7]. In the cerebellum, where PE level was unaltered, neither SELENOI mRNA nor protein expression was altered in ALS (Figure 2B,C).

### 3.3. Determining the Link Between SELENOI and TDP-43

**Figure 3:**
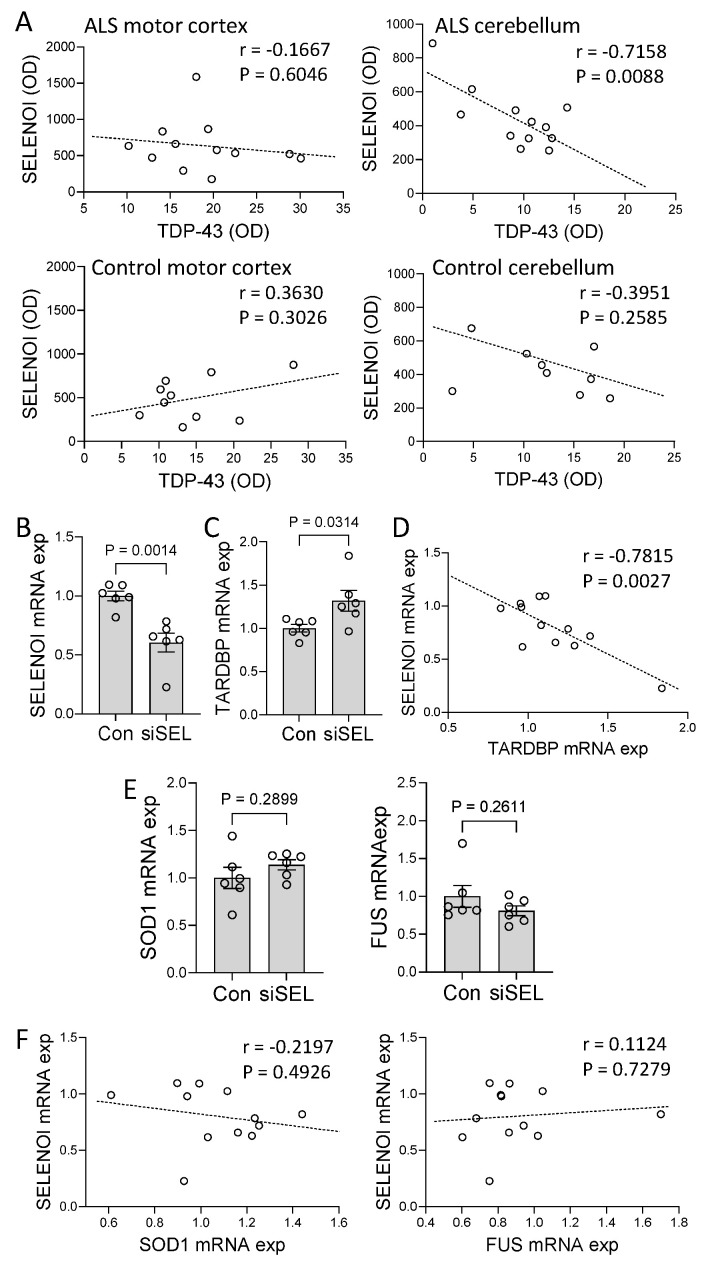
Effect of SELENOI on TARDBP expression. (**A**) Correlation between SELENOI protein expression and TDP−43 protein expression in amyotrophic lateral sclerosis (ALS) and control motor cortex and cerebellum. (**B**) SH−SY5Y neuronal cells were treated with SELENOI siRNA (siSEL) or scramble siRNA (control), and SELENOI mRNA expression measured by qPCR. (**C**) TARDBP mRNA expression was also measured in the cells. (**D**) Correlation between SELENOI mRNA expression and TARDBP mRNA expression in the cells. (**E**) Assessment of SOD1 and FUS mRNA expression in the cells. (**F**) Correlation between SELENOI mRNA expression and SOD1 and FUS mRNA expression in the cells. Data represent mean and S.E.M. as error bars.

It is unknown whether SELENOI is pathologically linked to TDP-43, the most important pathogenic protein in ALS brain. We therefore carried out a correlation analysis between SELENOI and TDP-43 in ALS brain using the two-tailed Pearson correlation at a confidence interval of 95%. There was a strong inverse correlation between SELENOI and TDP-43 in the cerebellum (Figure 3A). In contrast, this correlation was absent in the motor cortex (Figure 3A). The correlation was also absent in the control tissues (Figure 3A). To further investigate the relationship between SELENOI and TDP-43, SH-SY5Y neuronal cells were cultured and treated with SELENOI siRNA or scramble siRNA (control), and SELENOI and TARDBP expression were assessed by qPCR. Firstly, the knockdown of SELENOI expression in the SELENOI siRNA-treated cells was verified (Figure 3B). Importantly, the knockdown of SELENOI expression caused a significant upregulation of TARDBP expression (Figure 3C). Furthermore, SELENOI expression was inversely associated with TARDBP expression (Figure 3D), as was the case in ALS cerebellum (Figure 3A). In addition, we were interested in whether SELENOI affected other key genes associated with ALS, i.e., SOD1 and FUS. We found that the expression of SOD1 and FUS were unaffected by the SELENOI knockdown (Figure 3E) or associated with it (Figure 3F). When put together, these results suggest that SELENOI-mediated repression of TDP-43 is lost in ALS motor cortex, providing further evidence that SELENOI is dysregulated in ALS brain.

## 4. Discussion

Increasing evidence suggests that dysregulation of lipids contributes to the pathogenesis of ALS [5]. One of the prominent lipids in the human brain is phosphatidylethanolmine (PE), synthesized by the SELENOI gene. The importance of SELENOI and PE in brain function is underscored by the fact that mutations in SELENOI cause multiple and heterogeneous developmental and neurological dysfunctions, including delayed motor neuron development, delayed speech and reduced intellect [13]. However, how SELENOI contributes to ALS neuropathology was virtually unknown. To address this shortfall in knowledge, we carried out an analysis of the entire PE lipid class and SELENOI expression in the disease-affected motor cortex and disease-unaffected cerebellum of sporadic ALS and control brain. We found that 16 PE species and the total PE levels were significantly decreased in ALS motor cortex compared to controls. The PE levels were inversely correlated with disease duration only in the motor cortex. In contrast, no significant changes were detected in the cerebellum. Consistent with this, SELENOI was not correlated with TDP-43 in ALS motor cortex. When put together, these results suggest that SELENOI dysregulation may contribute to TDP-43 pathology in ALS brain.

Clues to understanding the contribution of SELENOI dysregulation in ALS pathogenesis come from studies carried out in a group of neurological disorders called hereditary spastic paraplegia (HSP) [22]. HSP is caused by loss-of-function mutations in the SELENOI gene [23]. These patients are typically characterized by abnormalities in neurodevelopment and neurodegenerative motor neurons that cause progressive weakness and contraction of the lower limbs, affecting mobility [23,24]. Some of these symptoms overlap with those of ALS. Although there are no reports of measurements of PE in HSP brain, LC-MS measurements of cell cultures of skin fibroblasts collected from HSP patients showed that eight PE species and the total PE were significantly decreased when compared to healthy controls [24]. Interestingly, PE 18:0p/18:1, one of the PE species that decreased in ALS motor cortex, was shown to be also decreased in the HSP cell cultures.

The recent generation of a conditional knockout mouse model, in which the SELENOI gene is inactivated only in the CNS, has shed light on the possible role of SELENOI in the context of ALS. The body weight of the SELENOI knockout mice was significantly lower compared to wild-type counterparts [15]. This is consistent with the reduced growth of humans with SELENOI mutations [23,24]. In ALS patients, weight loss is also common, with a clear association between body mass index and disease progression [25,26]. Another phenotypic similarity between the SELENOI knockout mice and ALS patients is the impaired voluntary movement and locomotion. Similar to limb stiffness and weakness in ALS patients, the SELENOI knockout mice displayed significant deficiency in the rotarod test and the vertical pole test, with the mice unable to grasp the vertical pole with their hindlimbs [15].

However, how SELENOI could be linked to TDP-43 pathology was not investigated in these mice. Nevertheless, motor dysfunction, similar to those of HSP and ALS patients, has already been linked to TDP-43 pathology [27]. Moreover, numerous studies have shown that overexpression of mutant TDP-43 causes neurodegeneration in animal models of ALS, including *Caenorhabditis elegans*, *Drosophila melanogaster*, zebrafish and mouse [28,29]. In humans, overexpression of wild-type TDP-43, as is the case in our ALS cohort, causes neurodegeneration in most (>95%) of ALS cases [30]. However, it was unknown how, if at all, SELENOI affects TDP-43 expression. We showed that SELENOI appears to repress TDP-43 expression, and this relationship is absent in ALS motor cortex. It is, however, unclear at this stage, whether the absence of SELENOI-TDP-43 relationship is due to compromised function or activity of SELENOI in ALS motor cortex. Nevertheless, this is an important finding as it provides a new avenue for controlling TDP-43 expression and subsequent TDP-43 accumulation in ALS neurons. The regulation of TDP-43 expression is thought to be, in part, autoregulated via binding of TDP-43 to the TDP-43 binding region (TDPBR) in the TARDBP 3′ UTR [31,32]. It is plausible to think that PE or PE derivatives in the nucleus membrane could be regulating the nucleocytoplasmic transport and diffusion of TDP-43 through the membrane. Alternatively, we speculate that PE or PE derivatives could be enhancing the binding of TDP-43 to the TDPBR and therefore increasing the repression of TDP-43 expression.

In terms of brain, the SELENOI knockout mice displayed marked changes in the lipid composition, with significant decreases in PE [15], which is consistent with the decreases in PE we observed in ALS motor cortex, wherein SELENOI dysregulation is implicated. Furthermore, the SELENOI knockout mice displayed an upregulation of glial fibrillary acidic protein (GFAP), indicative of reactive astrogliosis, in the dorsal striatum, the primary somatosensory cortex and the ventral posterior nucleus of the thalamus, the regions of the brain important in motor control [15]. Besides motor neuron degeneration, increased levels of GFAP and astrogliosis are also cardinal neuropathological features of ALS [33]. Astrocytes are important in maintaining and supporting neurons and they play multiple roles related to fluid and ion homeostasis, energy metabolism, synapse activity, and blood–brain barrier integrity [34]. However, in a disease state, activated astrocytes appear to exert a deteriorating effect on neuronal survival in ALS [35].

It was also interesting to note in the conditional SELENOI knockout mouse brain that PC was increased with a concomitant decrease in PE [15]. Likewise, in the brain of a *Drosophila melanogaster* model of ALS, PC was increased with a concomitant decrease in PE [10]. Consistent with these results, we found that PC was increased with a concomitant decrease in PE in ALS motor cortex. It was suggested that the increase in PC was a compensatory measure for the decrease in PE [15]. This compensatory relationship between PE and PC was also demonstrated in our in vitro study, in which neuronal cells were transfected with SELENOI cDNA, causing an increase in PE and a concomitant decrease in PC.

PE is a major component in cellular membranes, comprising ~15-25% of phospholipids in mammalian cells [36]. It plays three overarching roles in cellular context, as a structural component of cellular membranes, in tethering and sustaining the function of intrinsic proteins embedded in membranes, and as a precursor to other lipids, such as plasmenyl-PE. As a structural component of cellular membranes, PE contributes to shaping membrane curvature and regulating membrane rigidity and fluidity [37]. Plasmenyl-PE plays a number of critical roles in the brain, including inhibiting neurodegeneration and neuroinflammation and improving cognitive function [8]. In cultured cells of skin fibroblasts from HSP patients, plasmenyl-PE levels were decreased [24], as expected since its precursor PE was decreased in these cells. Consistent with this, plasmenyl-PE levels were decreased in HeLa cells with SELENOI deletion [24]. It is interesting to note that plasmenyl-PE is observed to be decreased in the postmortem brain tissues of other neurodegenerative and neurological diseases, including Alzheimer’s disease [38], Parkinson’s disease [39], Down syndrome [40] and schizophrenia [41], further suggesting the importance of plasmenyl-PE in neuronal development and function.

## 5. Conclusions

In summary, we produced some evidence that supports the idea that decreases in PE, as a consequence of SELENOI dysregulation, may cause changes to neuronal integrity and function, leading to neuropathology in ALS. We showed, for the first time, that SELENOI appears to repress TARDBP expression, and this relationship is absent in ALS motor cortex. These are important findings as they provide new insights into understanding how lipid dysregulation impacts on brain function and reveals new targets for controlling TDP-43 pathology in ALS. Future studies could involve testing our hypothesis in preclinical models of ALS, such as induced pluripotent stem-cell-derived neurons generated from ALS patients or mouse models of ALS.

## Figures and Tables

**Table 1:** Demographics of postmortem brain tissues from ALS and controls.

ID	Case	Age	Sex	Disease	Postmortem	Pathology	Cause of Death
		(yr)		duration (yr)	delay (hr)		
1	ALS	72	F	2	8	TDP-43	Respiratory failure
2	ALS	70	F	1	5	TDP-43	Cardiorespiratory failure
3	ALS	66	M	4	24	TDP-43	Cardiorespiratory failure
4	ALS	81	F	1	19	TDP-43	Cardiorespiratory failure
5	ALS	82	F	5	24	TDP-43	Cardiorespiratory failure
6	ALS	75	M	3	29	TDP-43	Multi organ failure
7	ALS	72	M	3	31	TDP-43	Cardiorespiratory failure
8	ALS	71	F	3	25	TDP-43	Respiratory failure
9	ALS	62	F	3	8	TDP-43	Motor neuron disease
10	ALS	64	F	1	27	TDP-43	Motor neuron disease
11	ALS	69	F	4	30	TDP-43	Motor neuron disease
12	ALS	78	F	2	24	TDP-43	Motor neuron disease
13	Control	85	F	-	23		Pneumonia
14	Control	79	M	-	8		Pulmonary embolism
15	Control	89	F	-	23		Metastatic adenocarcinoma
16	Control	93	F	-	7		Cardiovascular failure
17	Control	88	F	-	31		Congestive cardiac failure
18	Control	84	M	-	22		Cardiovascular failure
19	Control	84	M	-	36		Severe pulmonary hypertension
20	Control	80	F	-	29		Cardiac failure
21	Control	88	M	-	23		End-stage respiratory failure
22	Control	84	F	-	16		Papillary muscle rupture

## Data Availability

Data is available upon relevant ethical approval by contacting the corresponding author on reasonable request.

## Abbreviations

ALS	Amyotrophic lateral sclerosis
EPT1	ethanolamine phosphotransferase 1
HSP	hereditary spastic paraplegia
LC-MS	liquid chromatography-mass spectrometry
NfL	neurofilament light
PC	phosphatidylcholine
PE	phosphatidylethanolamine
SELENOI	selenoprotein I
TARDBP	TAR DNA binding protein
TDP-43	TAR DNA-binding protein 43
